# Using Deep Learning to Predict Minimum Foot–Ground Clearance Event from Toe-Off Kinematics

**DOI:** 10.3390/s22186960

**Published:** 2022-09-14

**Authors:** Clement Ogugua Asogwa, Hanatsu Nagano, Kai Wang, Rezaul Begg

**Affiliations:** 1 Institute for Health and Sport (IHES), Victoria University, Melbourne, VIC 8001, Australia; 2 University of Tsukuba, Tsukuba 305-8577, Japan

**Keywords:** minimum foot clearance, tripping prevention, falls prevention, deep learning, machine learning, gait biomechanics

## Abstract

Efficient, adaptive, locomotor function is critically important for maintaining our health and independence, but falls-related injuries when walking are a significant risk factor, particularly for more vulnerable populations such as older people and post-stroke individuals. Tripping is the leading cause of falls, and the swing-phase event Minimum Foot Clearance (MFC) is recognised as the key biomechanical determinant of tripping probability. MFC is defined as the minimum swing foot clearance, which is seen approximately mid-swing, and it is routinely measured in gait biomechanics laboratories using precise, high-speed, camera-based 3D motion capture systems. For practical intervention strategies designed to predict, and possibly assist, swing foot trajectory to prevent tripping, identification of the MFC event is essential; however, no technique is currently available to determine MFC timing in real-life settings outside the laboratory. One strategy has been to use wearable sensors, such as Inertial Measurement Units (IMUs), but these data are limited to primarily providing only tri-axial linear acceleration and angular velocity. The aim of this study was to develop Machine Learning (ML) algorithms to predict MFC timing based on the preceding toe-off gait event. The ML algorithms were trained using 13 young adults’ foot trajectory data recorded from an Optotrak 3D motion capture system. A Deep Learning configuration was developed based on a Recurrent Neural Network with a Long Short-Term Memory (LSTM) architecture and Huber loss-functions to minimise MFC-timing prediction error. We succeeded in predicting MFC timing from toe-off characteristics with a mean absolute error of 0.07 s. Although further algorithm training using population-specific inputs are needed. The ML algorithms designed here can be used for real-time actuation of wearable active devices to increase foot clearance at critical MFC and reduce devastating tripping falls. Further developments in ML-guided actuation for active exoskeletons could prove highly effective in developing technologies to reduce tripping-related falls across a range of gait impaired populations.

## 1. Introduction

**Figure 1:**
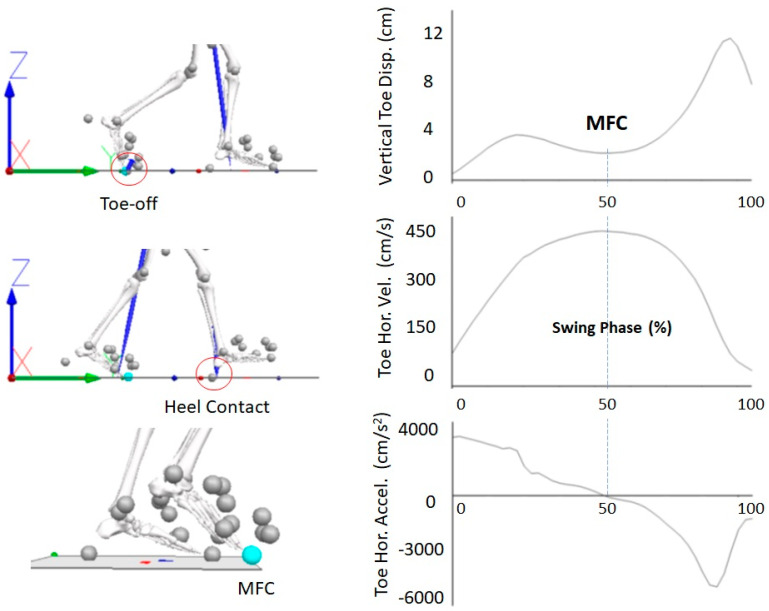
(**Left-top**) toe-off, (**left-middle**) heel contact, and (**left-bottom**) Minimum Foot Clearance (MFC) events; marker setup for foot modelling infrared emitting diodes (IREDs) and virtual markers; MFC, the intermittent event between toe-off and heel contact. (**Right**) Swing-phase kinematics showing: (**right-top**) MFC detection at the local mid-swing minimum vertical displacement, (**right-middle**) MFC coincident with maximum horizontal velocity, (**right-bottom**) MFC timing at zero horizontal acceleration.

Falls are a major cause of serious injury during locomotion, particularly for populations with impaired gait function. Approximately one in three people over 65 years fall annually, and 9–20% of cases are associated with critical injuries [1,2]. Pathological populations have an even higher risk of falls; for example, 50% of stroke survivors and 68% of Parkinson’s patients fall at least once a year [3,4]. The leading cause of falls is tripping, accounting for up to 53% of cases [5,6]. Tripping can be defined biomechanically as unanticipated foot contact with the walking surface, or an object on it, generating an impact sufficient to destabilise the walker [7]. Minimum Foot Clearance (MFC) is the gait event at which tripping risk is greatest [8] and, as shown in Figure 1 (right), MFC can be identified by computing the vertical position of the swing foot (i.e., the toe) relative to the floor using 3D motion capture data [9]. 

Declines in neuromuscular function reduce their capacity, affecting mid-swing foot–ground clearance [10], and interventions to prevent tripping, such as exercise programmes [11] and shoe-insole modifications [12,13], have been designed to increase mid-swing vertical displacement and reduce step-to-step MFC variability. Reducing MFC height variability is important in decreasing the probability of very low foot–ground clearances, represented by higher frequencies in the low-clearance region of the MFC distribution. Recently, real-time biofeedback-training interventions to increase MFC height and reduce variability have been trialled using a real-time display of the foot’s vertical displacement at MFC presented on a computer monitor during treadmill walking [14]. This technique was successful at improving swing foot clearance with unilateral post-stroke individuals, but a major limitation of biofeedback-training rehabilitation is the dependency on extended, clinic-based rehabilitation requiring a 3D motion capture system to record lower limb motion [15]. Techniques to improve swing foot control that can be used widely outside the laboratory will confer considerable advantages, in terms of cost effectiveness and an increased range of gait-rehabilitation applications suitable for everyday settings. 

Delfi et al. [16,17] reviewed sensor technologies for MFC detection suitable for integration into wearable assistive devices; when considering cost and ease of use, inertial measurement units (IMUs) were the preferred application. Linear acceleration and angular velocity can be recorded from IMUs, and by double integrating the acceleration data (m/s^2^), the critical foot displacement (m) can be computed. A remaining challenge for accurately estimating lower limb segment displacement-time characteristics, however, is the ‘drift’ associated with error accumulation over time [18]. While the accuracy of IMU-based approaches can be improved using machine learning prediction algorithms [19], they still lack the millimetre precision needed for reliable MFC estimation, given a range of typically only 1–2 cm. While less precise kinematic event estimates may not be so problematic, for gait parameters with greater magnitude, such as stride length [20], they are not suitable for estimating the foot trajectory measurements needed to guide intelligent actuator control systems, and alternative approaches are urgently required.

In addition to the problem of recording limb motion in real-world settings, we are investigating gait biomechanics techniques to enable real-time prediction of critical gait cycle events, such as MFC. These innovations will be extremely powerful in equipping active exoskeletons with real-time limb trajectory correction. If, for example, a wearable ankle exoskeleton is equipped to predict MFC, the swing foot can be elevated by providing additional ankle dorsiflexion with the optimal timing, thus reducing tripping risk [21]. 

## 2. State of the Art, Research Question, Hypothesis

**Figure 2:**
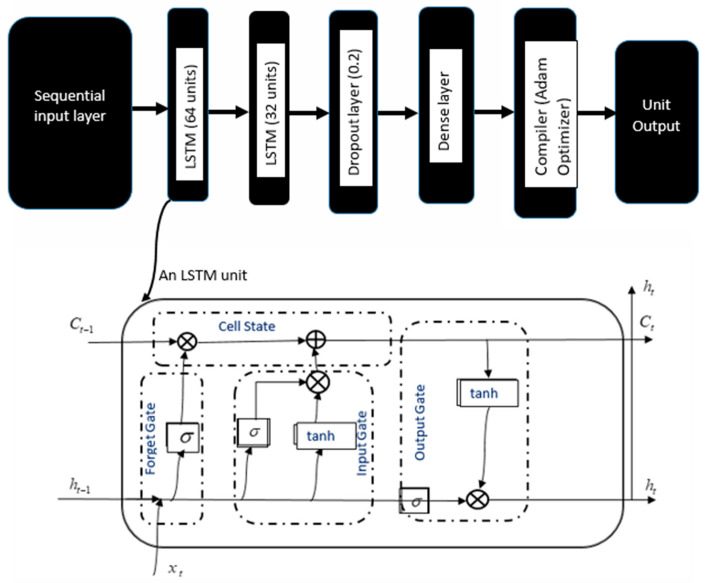
LSTM architecture with two LSTM layers (64 and 32 units each) stacked together followed by a drop-out layer to avoid overfitting, a dense layer, and a compiler. Below it is a representation of a unit LSTM architecture consisting of Forget gate to decide what must be removed from the ($h_{t-1}$) state, the Input gate to write from present input to current cell state, and the Output gate to decide what to output from cell state using the sigmoid function. The outputs of the Input and Forget Gate are summed together to determine each current Cell State.

The research question addressed in this experiment was whether MFC timing could be predicted from the foot’s linear acceleration and angular velocity at the preceding toe-off. Toe-off marks the transition from stance, when the foot is in contact with the ground, to swing, approximately 0.2 s prior to MFC [10,22,23], providing sufficient time for device actuation. Despite the complexity of swing foot clearance patterns, MFC usually coincides with maximum toe velocity and, in principle, near-zero linear acceleration and angular velocity (Figure 1, right). Advances in machine learning have significantly improved research capabilities and our understanding of human gait. Figure 2 is a simplified long short-term memory (LSTM) cell architecture, a type of artificial neural network that uses dependencies in sequence to make predictions. LSTMs have been shown to have various important applications such as predictions in human movement biomechanics [24], gait recognition with machine learning [25], and gait classification with artificial neural network [26]. The research aims were to devise algorithms to predict MFC event. If MFC timing can be accurately predicted, interventions based on assistive devices (e.g., active ankle exoskeleton) could, for example, target increasing swing foot clearance to reduce tripping falls. It was hypothesised that toe-off characteristics would reliably predict MFC timing from kinematic information at toe-off. 

## 3. Materials and Methods

### 3.1. Participants 

Participants were 13 healthy young (24.2 ± 3.2 years) adults recruited from the Victoria University community; all were free of injuries or health conditions that may have affected their gait. Physical characteristics were height (1.77 ± 0.07 m) and body mass (77.2 ± 14.5 kg). The Victoria University Human Research Ethics Committee approved the experimental protocol, and all participants voluntarily completed the informed consent procedures prior to participation.

### 3.2. Experimental Procedures and Data Collection

Three Optotrak (Optotrak^®^, NDI, Waterloo, ON, Canada) motion capture cameras surrounding an 8-metre laboratory walkway sampled the motion of Infrared Light Emitting Diodes (IREDs) at 100 Hz. Two force plates (AMTI) positioned midway along the walkway recorded foot–ground reaction forces (GRF) at 1000 Hz. Each participant completed a minimum of 60 step cycles at preferred walking speed. 

**Figure 3:**
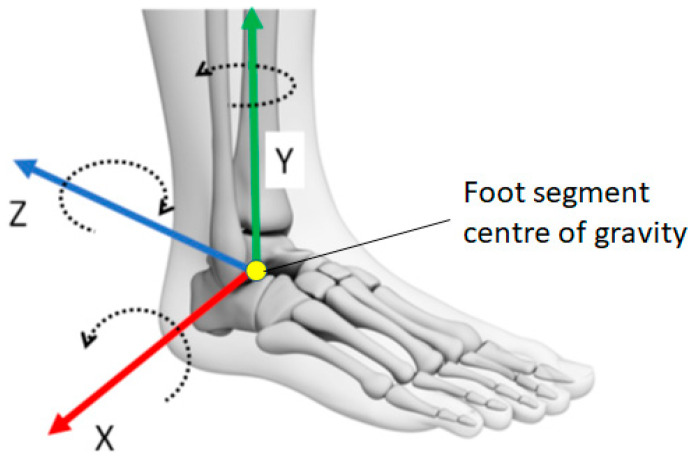
Definitions of foot kinematics data based on the segment coordination system. X, Y, Z in line with linear acceleration (+), arrows around the axes indicating positive (+) angular velocity direction.

The motion capture three-dimensional (3D) position coordinates were low-pass filtered (6 Hz) prior to kinematic analysis. Occluded data of up to 10 frames were interpolated using ±3 frames of the missing section using a third-order polynomial estimation. Virtual marker functions (Visual 3D, C-motion, Inc., Germantown, MD, USA) based on the cluster technical markers attached anterior to the toe and mid-foot defined the anatomical reference frame and modelled the foot segment and associated kinematic data. The foot complex was constructed using the following anatomical landmarks: heel (the proximal end of the foot), the 2nd and 5th metatarsal heads, toe (the most anterior and superior surface of the foot), and lateral and medial malleolus [27]. Foot segmental kinematic data including tri-axial linear accelerations and angular velocities were obtained throughout the swing-phase, consistent with the local coordinates illustrated in Figure 3. 

### 3.3. MFC, Heel Contact and Toe-Off Event Definitions and Machine Learning Inputs

As in Figure 1, the swing-phase was determined as the period between toe-off and heel contact defined, respectively, by the vertical ground reaction force (GRF) offset and onset at 10 N thresholds [28]. Minimum foot clearance (MFC) was defined as the lowest vertical displacement of the swing toe during the mid-swing-phase, coincident with maximum horizontal toe velocity and zero acceleration (Figure 1, right). Table 1 summarises the mean and standard deviation (SD) of the three gait event labels, heel contact, toe-off and MFC, respectively, encoded as 2, 1 and 0. All tri-axial linear accelerations and angular velocities were relative to the foot segment’s centre of gravity, similar to the method adopted by De Witt et al. [29] for determining toe-off timing from heel stride. 

### 3.4. Neural Network Architecture

#### 3.4.1. Background and Model Design

Long short-term memory (LSTM) networks are recurrent neural networks designed to apply dependences in sequence prediction, using cell states that learn the order of dependents for sequential tasks. These sequences are periodic events that can be converted into digital time-series representations suitable for human gait prediction [24,30]. The LSTM-based regression model implemented in this work was designed using the Python programming language (Python Software Foundation, Python Language Reference, version 3) [31], scipy [32], pandas [33], Keras APIs [34] and TensorFlow 2 [35] with a Keras back end. The dataset included 6 kinematic feature variables from translation along linear accelerations (CGAccX, CGAccY, CGAccZ) and angular velocities (AngVelX, AngVelY, AngVelZ), and their corresponding labels (heel stride, toe-off, and minimum foot clearance as (2,1,0), respectively, using Keras LabelEncoder) were the 7th feature (Table 1). The training and validation datasets were transformed into a sequence of input/output samples of 7 input features representing heel contact, toe-off and MFC in this sequence. We used rolling cross-validation which requires every immediate past data to predict the next future so that each n-future is first predicted and then put back as part of training set before the next n-future is predicted. Average performance was reported across the different folds based on the MAE metric. The labels were encoded to translate the event tags into integers (Table 1), with the dataset normalised to zero mean and unit standard variance using RobustScaler. The final model was designed as follows: The first LSTM layer with output shape (None, 50, 64) followed by a hidden layer of units 32, a drop-out layer, and an output dense layer of unit 1. The network was compiled using an Adam optimizer (learning rate 0.001) and the activation of all LSTM layers was set to a rectilinear unit (ReLU) function. 

#### 3.4.2. Evaluation and Performance Metrics 

The input sliding window was fixed at 50 timesteps and trained for 50 epochs throughout. The output window prediction was tested for 10 timesteps (0.1 s) for the 7 feature variables. To assess the model’s performance a Huber loss function was used to compute loss minimisation during training. The error margin for MFC timing prediction based on toe-off characteristics was investigated using the LSTM-based regression model with MAE as the metric.

## 4. Results

**Figure 4:**
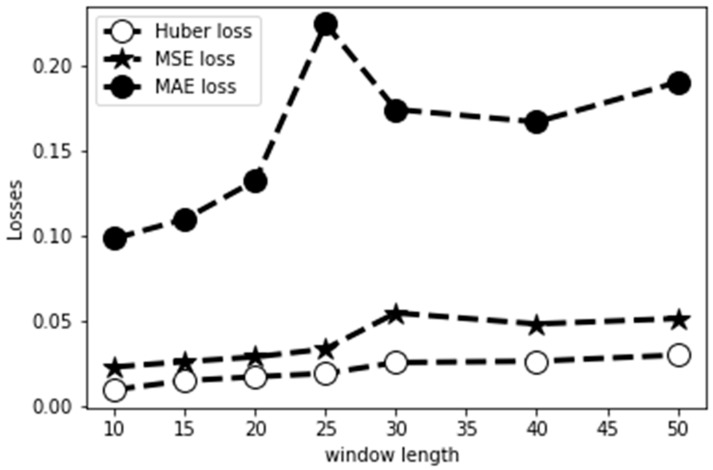
Performance comparison of Huber loss, MSE loss, and MAE loss functions on the training data with 50 past observations at different window lengths.

**Figure 5:**
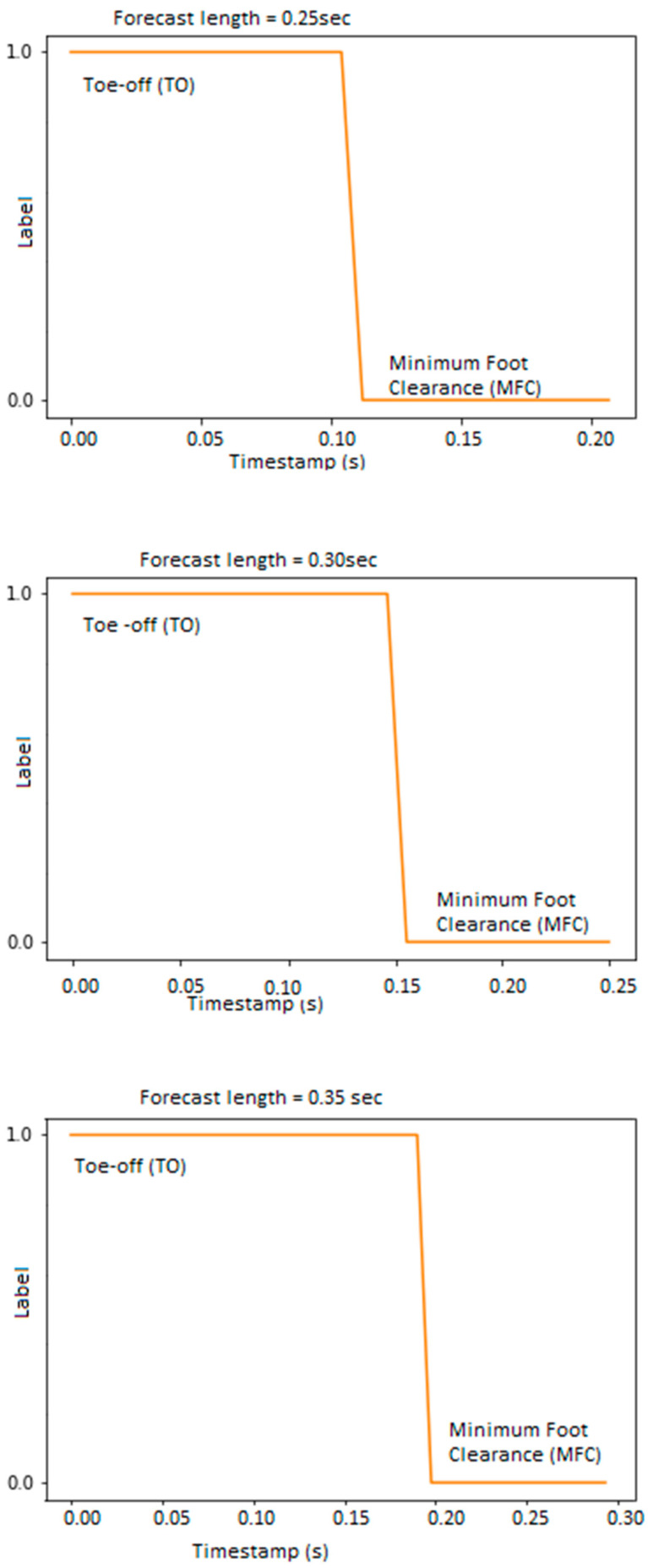
MFC timing forecast from toe-off kinematics at five prediction horizons between 0.15 s and 0.35 s. MFC forecasting diminishes and transitions to a new cycle as the forecast horizon increases.

Figure 4 shows the loss variation for a range of window lengths, indicating that model training with the Huber loss function showed better prediction of MFC timing within the swing-phase than the MSE and MAE regression functions. MAE, MSE and Root Mean Squared Error (RMSE) are statistical metrics used in Keras API to evaluate regression model performance by calculating the distance between predictions and the ground truth. MSE is the mean square difference between the target value and the regression model predictions, while MAE is simply the mean absolute difference between the ground truth and the predicted values. MAE was used to determine model prediction accuracy because it is considered to be more robust in terms of accommodating outliers, does not amplify errors, and quantifies the discrepancy between predictions and output (Figure 5).

When the training algorithm model employed 50 prior observations, the predictions obtained from 0.25 s to 0.35 s were consistent with previous studies reporting that the interval between toe-off and MFC for healthy young adults is typically 0.2 s [22,23]. The MFC timing forecast began 0.15 s after toe-off, peaked after 0.2 s, and gradually diminished as the forecast horizon increased (Figure 5). In summary, the results showed that MFC timing forecasts relative to toe-off were unreliable beyond the hypothesised 0.35 s upper boundary and the current algorithm functioned with acceptable accuracy within the typical 0.25–0.35 s between toe-off and MFC. 

## 5. Discussion

The aim of this study was to investigate the capability of an artificial neural network to predict MFC timing on the basis of toe-off linear acceleration and angular velocity. Previous studies have focused on gait phase classification and MFC height estimation using double integration of acceleration to compute the vertical displacement for various gait modes, such as grade walking (ascent, descent and level), both overground and on a treadmill [24]. Our approach took advantage of pre-programmed 3D motion capture data (Optotrak) to automatically label swing-phase gait events, including toe-off, MFC, and heel contact. This procedure was similar to the radial basis function network (RBFN) approach used by Miyake et al. [36] to predict toe clearance characteristics. 

The results showed that the six tri-axial feature variables, linear acceleration (x, y, z) and angular velocity (x, y, z), could be used to reliably classify swing-phase events and, specifically, predict MFC timing. Huber regression functions provided consistently low loss, even when the window length was considerably increased [37]. When using Deep Learning error estimation, at each iteration, it is essential to maintain prediction accuracy. The Huber loss function incorporates the advantages of mean squared error (MSE) and mean absolute error (MAE) computations to provide robust learning in deep regression tasks [38,39]. We found that using a Huber loss function for training with MAE as the accuracy metric, our subsequent prediction of MFC was practicable at least 0.15 s later with a mean absolute error of 0.07 s. The current Deep Learning algorithm may, therefore, not be applicable to faster-paced walking, in which the MFC may occur sooner than 0.15 s after toe-off, or conversely, to slower walking, with longer than 0.35 s between the two events. While this prediction interval is likely to be acceptable for healthy, preferred speed walking, further data feeding is necessary in order to be able to accommodate atypical gait patterns with respect to the period between toe-off and MFC. Faster prediction features may also be useful, and could be achieved by increasing the data volume, leading to more timely actuation of the active assistive device. Such Deep Learning algorithms can be incorporated into active exoskeleton control to support pathological gait rehabilitation, using training data from specific gait impaired populations. 

Falls often result in serious injuries for vulnerable populations, and successful tripping risk management can contribute to fall prevention [1,2,5,6]. Minimum foot clearance (MFC) is the critical swing-phase event during which tripping is likely to result in forward falls [40]. Although 3D motion capture systems have been used for micro-measurement of MFC height and timing, everyday application requires the prediction of MFC timing on the basis of the preceding gait event (i.e., toe-off). If such techniques are successfully developed, wearable sensors could be used to predict forthcoming MFC timing using a feedforward mechanism, allowing real-time intervention by an assistive device. 

In biomechanical experiments, 3D motion capture is considered to be the ‘gold standard’, and the purpose of the current study was to investigate whether foot segmental kinematic information at toe-off would predict the timing of MFC. The selected inputs were linear acceleration and angular velocity, assuming that the next phase of the current research will be their application in IMUs. As indicated earlier, if ankle active exoskeletons [41] incorporate such feed-forward prediction capacity and are customised to directly support walking, dorsiflexion can be effectively actuated at MFC timing, estimated from toe-off characteristics determined by IMUs. This is expected to reduce the risk of tripping by providing sufficient MFC, but it can also possibly assist the wearer with learning the optimum ankle motion during the swing phase. Integration of walk-assist functions into ankle exoskeletons may be the reliable solution to reduce the tripping risk. 

Feedforward prediction of MFC from toe-off kinematics can be incorporated into other assistive devices. Electrical stimulators for dorsiflexors have been previously used with a footswitch installed at the heel to trigger electrical stimulation between heel-off and next heel contact [42]. In healthy gait, however, dorsiflexors should be active primarily around the MFC and the heel contact of the swing phase [43,44]. The current feedforward approach can therefore also benefit this technology in terms of providing the stimulus at the right time (i.e., MFC and heel contact). Application into smart footwear could be another practical development of the current concept. While IMU-based smart systems can measure the foot orientation and kinematic information, detection of accurate MFC timing is quite challenging if height measurements are used based on double-integration of acceleration data. Attempts at direct MFC measurement still has an error of 26%, with a 7 mm discrepancy being observed at an MFC height of approximately 26.8 mm, which could add further drifts in predicting the timing of MFC [19]. Higher accuracy in predicting the MFC event was identified using the current concept focusing on the temporal approach based on foot segment kinematics, and could be more feasible for real-time MFC detection. Electric powered ankle-foot devices have been developed to detect gait intents with EMG signals [41]; all such devices can have ML algorithms incorporated to reduce tripping falls by means of biofeedback information.

Nevertheless, future research efforts should equally be devoted to MFC height measurement based on the double integration of vertical swing foot displacement between toe-off and MFC [18,19].

## 6. Conclusions

Our results showed that MFC timing can be predicted from toe-off characteristics using tri-axial angular velocities and linear accelerations with deep neural network. The next phase of our work will be to apply the current concept using inertial measurement units (IMUs) that can be attached to ankle active exoskeletons to provide adequate mechanical support at MFC. Further validation is necessary to incorporate a wider range of populations (e.g., pathological populations, senior adults) to achieve reliable and generalisable MFC prediction algorithms with a limited error margin.

## Figures and Tables

**Table 1:** Mean and standard deviation of the distribution of the dataset for each label; CGAcc = foot centre of gravity acceleration, AngVel = angular velocity of foot centre of gravity, SD = standard deviation, HC = heel contact, TO = toe-off and MFC = minimum foot clearance.

Event Label	Encode		CG AccX (m/s²)	CG AccY (m/s²)	CG AccZ (m/s²)	Ang VelX (°/s)	Ang VelY (°/s)	Ang VelZ (°/s)
HC	2	Mean	−0.82	13.35	−4.39	−1.74	0.63	−0.17
		SD	5.10	11.00	9.15	3.33	1.22	1.00
TO	1	Mean	0.29	14.36	−1.14	−5.73	−0.25	0.46
		SD	3.79	5.21	6.01	3.83	0.98	1.07
MFC	0	Mean	−0.12	5.09	−0.14	5.70	0.41	−0.23
		SD	2.73	2.45	4.73	0.78	0.62	0.79

## Data Availability

Data are available on reasonable requests.
